# Cloning and plant‐based production of antibody MC10E7 for a lateral flow immunoassay to detect [4‐arginine]microcystin in freshwater

**DOI:** 10.1111/pbi.12746

**Published:** 2017-06-05

**Authors:** Stanislav Melnik, Anna‐Cathrine Neumann, Ryan Karongo, Sebastian Dirndorfer, Martin Stübler, Verena Ibl, Reinhard Niessner, Dietmar Knopp, Eva Stoger

**Affiliations:** ^1^ Department of Applied Genetics and Cell Biology University of Natural Resources and Life Sciences Vienna Austria; ^2^ Institute of Hydrochemistry and Chair for Analytical Chemistry Technical University Munich Munich Germany

**Keywords:** molecular farming, lateral flow immunoassay, plant‐made antibody, microcystin, water contamination

## Abstract

Antibody MC10E7 is one of a small number of monoclonal antibodies that bind specifically to [Arg4]‐microcystins, and it can be used to survey natural water sources and food samples for algal toxin contamination. However, the development of sensitive immunoassays in different test formats, particularly user‐friendly tests for on‐site analysis, requires a sensitive but also cost‐effective antibody. The original version of MC10E7 was derived from a murine hybridoma, but we determined the sequence of the variable regions using the peptide mass‐assisted cloning strategy and expressed a scFv (single‐chain variable fragment) format of this antibody in yeast and a chimeric full‐size version in leaves of *Nicotiana tabacum* and *Nicotiana benthamiana* to facilitate inexpensive and scalable production. The specific antigen‐binding activity of the purified antibody was verified by surface plasmon resonance spectroscopy and ELISA, confirming the same binding specificity as its hybridoma‐derived counterpart. The plant‐derived antibody was used to design a lateral flow immunoassay (dipstick) for the sensitive detection of [Arg4]‐microcystins at concentrations of 100–300 ng/L in freshwater samples collected at different sites. Plant‐based production will likely reduce the cost of the antibody, currently the most expensive component of the dipstick immunoassay, and will allow the development of further antibody‐based analytical devices and water purification adsorbents for the efficient removal of toxic contaminants.

## Introduction

Microcystins are nonribosomal cyclic heptapeptides produced by several species of freshwater cyanobacteria. They are powerful, tumour‐promoting hepatotoxins that can pose significant health risks to animals and humans due to their remarkable stability in the environment and their ability to enter into the food chain. Exposure can therefore occur directly, via the consumption of contaminated water from eutrophic water bodies affected by algal blooms, or indirectly through the consumption of freshwater fish and seafood (Poste *et al*., 2011). Microcystin–leucine–arginine (MC‐LR) is the most widespread and toxic microcystin congener. It can induce liver and lung damage (Robinson *et al*., 2015), or disrupt gene expression and DNA replication (Hehle *et al*., 2015), and it also acts as a tumour promoter (Fujiki and Suganuma, 2011; Xu *et al*., 2013). The World Health Organization recommends that MC‐LR levels (free plus cell bound) should be limited to 1 μg/L in drinking water (WHO, 1998). In times of climate change and agricultural intensification, the distribution of cyanobacteria in inland waters has greatly increased even in temperate regions over the last few years, and improved concepts for monitoring and decontamination/purification are required (Ibelings *et al*., 2016; Ibrahim *et al*., 2016; Moe *et al*., 2016).

Several physical, chemical and biological methods have been developed or proposed for the detection and removal of microcystins (Bialczyk *et al*., 2014; El Semary, 2010; Tippkötter *et al*., 2009). These include nonselective techniques that remove organic compounds from water, such as adsorption to carbon, and the use of more selective chemical polymers that can be produced by molecular imprinting (Le Noir *et al*., 2006, 2007). Nevertheless, each of these methods has limitations in terms of specificity, efficiency or the requirement of large quantities of analyte for the synthesis of technically relevant quantities of polymer. Thus, there remains a need for highly selective and cost‐effective concepts in water purification.

Antibodies are suitable for the specific detection and removal of cyanotoxins due to their unparalleled sensitivity and selectivity. The most promising antibody for the selective binding of [Arg4]‐microcystins is currently MC10E7, a murine monoclonal IgG1 with mid‐point values for MC‐LR of 0.06 μg/L (Zeck *et al*., 2001). This antibody has been used to survey natural water sources and food samples (Gurbuz *et al*., 2009, 2016). However, its wider application for the development of inexpensive express test systems or water purification adsorbents (biofilters) is hampered by the high cost and limited production capacity inherent to hybridoma‐based expression systems. In addition, hybridoma cultures can become unstable over time, leading to a loss of antibody productivity (Coco‐Martin *et al*., 1992). The situation could be significantly improved by the use of heterologous production systems. This requires the rescue of variable region sequences that determine the unique epitope specificity and affinity, but such rescued sequences can then be used to build diverse recombinant antibody formats including full‐size chimeric antibodies with constant regions of antibody chains from various species and single‐chain variable fragments (scFvs) (Toleikis *et al*., 2004). Small antibody derivatives such as scFvs are routinely produced in microbial systems because they fold spontaneously and do not usually require extensive post‐translational modifications. In contrast, full‐size antibodies contain multiple disulfide bonds and N‐glycans, and usually mammalian cells such as Chinese hamster ovary (CHO) cells are the favoured production platform because they produce high titres (Kunert and Reinhart, 2016). However, the maintenance of mammalian cells is rather expensive making it worthwhile to explore alternative production platforms for low‐margin antibodies used as technical reagents (Frenzel *et al*., 2013). Yeast is less costly and has the capacity to perform post‐translational modifications, but has mainly been employed for the production of antibody fragments, while only limited data concerning full‐size IgG expression are available (Frenzel *et al*., 2013; Gasser *et al*., 2006). Plants on the other hand offer the necessary economy and scalability and have been used extensively for the production of antibodies, including complex full‐size formats (Ma *et al*., 1995; Orzaez *et al*., 2009; Sack *et al*., 2015). While some antibodies are intended to function in the plant itself, for example to protect the plant against pathogens or to modulate signalling pathways (Safarnejad *et al*., 2011), most antibodies expressed in plants are destined to be purified in compliance with good manufacturing practice (GMP) for medical applications (Stoger *et al*., 2014). The scalability of plant‐based expression systems has been successfully demonstrated by the development of automated production facilities for the rapid manufacture of protein pharmaceuticals in plants at industrial scales (Holtz *et al*., 2015; Wirz *et al*., 2012). A tobacco‐derived antibody was approved by Cuban authorities in 2006 as an affinity purification reagent for a hepatitis B vaccine produced in yeast (Pujol *et al*., 2005). Although this antibody is not used as a pharmaceutical, it nevertheless had to meet GMP standards as part of the vaccine manufacturing process. Another tobacco‐produced antibody that prevents dental caries (CaroRX) has received market approval as a medical device, and the experimental drug ZMapp, a combination of three humanized monoclonal antibodies that recognize an Ebola virus surface glycoprotein, was manufactured by transient expression in *Nicotiana benthamiana* plants by Kentucky BioProcessing under licence from Mapp Biopharmaceuticals Inc. San Diego, CA, USA (Qiu *et al*., 2014). A number of further plant‐derived antibodies for pharmaceutical application have entered into clinical studies (Group and Multi‐National, 2016; Ma *et al*., 2015; Stoger *et al*., 2014).

In contrast to pharmaceuticals, plant‐derived nonpharmaceutical proteins have the advantage that they can reach the market faster because of the much lower regulatory burden. The current commercial nonpharmaceutical product portfolio ranges from technical enzymes, diagnostics and research‐grade reagents to cosmetic products (Fischer *et al*., 2013; Paul *et al*., 2013), and overall, there are more nonpharmaceutical products already on the market than plant‐derived pharmaceutical proteins (Tschofen *et al*., 2016). Just like pharmaceutical products, nonpharmaceutical products also benefit from the low costs and greater scalability of plants, but the benefits are magnified because downstream processing and purification do not have to meet the strict criteria enforced for pharmaceutical GMP. The production of nonpharmaceutical antibodies in various formats has been demonstrated for applications in diagnostics, food processing and quality validation, but none of these products have yet reached the market (De Meyer *et al*., 2014; Ritala *et al*., 2014).

However, low‐cost antibodies directed against environmental pollutants would have great potential for use in biosensors and purification matrices. Antibodies directed against environmental pollutants have been produced in plants (Barbi *et al*., 2011; Drake *et al*., 2010) aiming to protect the plant against toxins and for applications in phytoremediation or the *in situ* cleaning of contaminated sites, but antibodies have not yet been purified from plants for environmental applications such as the development of bioanalytical devices or filters for the capture of cyanotoxins.

Here, we describe the cloning and overexpression of recombinant variants of MC10E7 in plant and yeast expression systems. Leaves of *Nicotiana tabacum* and *N. benthamiana* appear suitable for the rapid production of large quantities of the full‐size recombinant antibody. A comparison of the plant‐derived antibody and its original hybridoma‐derived counterpart confirmed that the antigen‐binding properties of MC10E7 are retained in the heterologous expression system allowing the development of a lateral flow immunoassay and similar applications.

## Results

### Antibody V‐gene rescue by peptide mass‐assisted cloning

**Figure 1:**
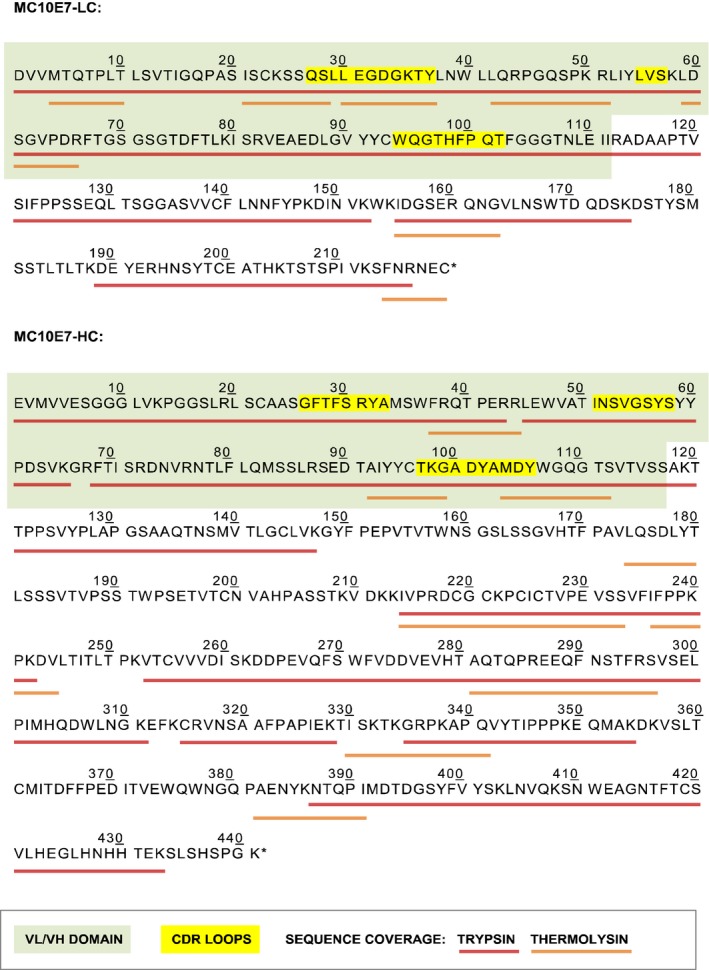
PMF sequence coverage of the hybridoma‐derived MC10E7 mAb (MALDI‐TOF‐MS).

The antibody heavy chain (HC) and light chain (LC) cDNAs were isolated by the reverse transcription of total RNA from the MC10E7 hybridoma cell line followed by 5′‐RACE and the ligation and cloning of the cDNA products. Individual colonies were then screened by colony PCR and sequencing. Sequences from at least five independent clones of the heavy and light chains were aligned (Figure S1) and theoretical peptide mass fingerprints (PMFs) of the consensus sequences were compared to the observed PMFs of the hybridoma‐derived MC10E7 (Figure 1). For accurate PMF annotation, the complete heavy and light chain cDNA sequences were used. Wrong, nonfunctional or aberrant antibody transcript sequences were excluded. We particularly encountered this problem during isolation of the LC sequence from the MC10E7 hybridoma cell line, which happened to contain a second light chain transcript with an unrelated but otherwise normal variable region sequence (data not shown).

Clones that matched the predicted sequences in both the light and the heavy chain variable domains (VL and VH, respectively) were selected for the construction of expression vectors. The cDNA sequences corresponding to each antibody chain are shown in Figure S1.

### Recombinant antimicrocystin antibodies can be produced in heterologous yeast and plant expression systems

The antibody was first evaluated in its more compact scFv format, and for this, the methylotrophic yeast *Pichia pastoris* was chosen as an expression platform. Small‐scale cultures were prepared in shake flasks, and antibody expression was achieved using the endogenous methanol‐inducible *AOX1* promoter. The best yield was ~4 mg of the fully soluble purified scFv per litre of medium which contained 36 g fresh weight (FW) of yeast cells at harvest. Size fractionation by polyacrylamide gel electrophoresis revealed that a significant fraction of the scFv polypeptides had slightly higher molecular weight than predicted (Figure S2). This phenomenon has been observed by others and can be attributed to imprecise α‐factor signal peptide cleavage (Ettayebi and Hardy, 2008). The antigen‐binding activity of the scFv was ~16‐fold lower than that of the full‐length hybridoma IgG; that is, the inflection point (IC_50_ value) of the ELISA calibration curve of only 0.701 ± 0.33 μg/L was obtained. We tested scFv variants with two different VL‐VH linkers (VL‐(G4S)_3(4)_‐VH), but there was little difference in performance.

**Figure 2:**
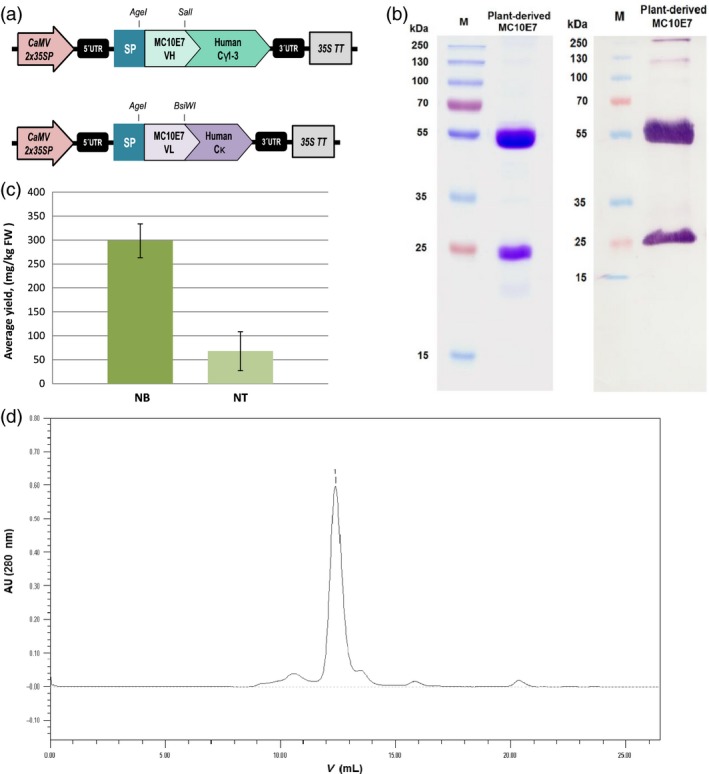
(a) Vector cassettes used for transient overexpression of the chimeric MC10E7 in plant leaves. The VL and VH sequences of MC10E7 were used to reconstruct a full‐size antibody based on constant regions of human IgG1. To express full‐size antibody, genes for the heavy and light chains were codelivered to plant cells on separate binary plasmids. CaMV2x35SP, enhanced cauliflower mosaic virus (CaMV) 35S promoter; 35S TT, cauliflower mosaic virus (CaMV) 35S terminator; UTR, untranslated region of the tobacco etch virus; SP, murine IgG signal sequence for entry into the endoplasmic reticulum; Cγ1‐3, human IgG1 constant domain for the heavy chain; Cκ1 human constant domain for the kappa light chain; VH/L region, variable region of the MC10E7 antibody heavy or light chain. (b) Left panel: SDS‐PAGE of the purified recombinant plant‐produced antimicrocystin antibody MC10E7 (5 μg) under reducing conditions. Staining with Coomassie Brilliant Blue R‐250 showing the heavy and light chains; right panel: immunoblot of the same sample (≥100 ng) probed with anti‐human IgG (H + L)‐AP conjugate. (c) Comparison of the average yields of recombinant full‐size antimicrocystin mAb achieved in *Nicotiana benthamiana* (NB) and in *Nicotiana tabacum* SR1 (NT) leaves after Protein G purification. Data from five independent infiltration experiments on each plant species. Error bars represent SD. (d) Size‐exclusion chromatography of the Protein G purified plant‐produced MC10E7. Main peak (1) corresponds to MC10E7; smaller peaks – protein impurities.

The VL and VH sequences were therefore used to reconstruct a full‐size antibody by fusing them to the constant regions of human IgG1 antibody chains proven to express well in *N. benthamiana* (Kapelski *et al*., 2015). We evaluated *N. benthamiana* and *N. tabacum* L. cv. Petit Havana SR1 as expression platform for the chimeric antibody (Figure 2). Initial evaluation involved a small‐scale transient expression experiment in which the antibody heavy and light chains were codelivered with the *Tomato bushy stunt virus* (TBSV) p19 suppressor of post‐transcriptional gene silencing (Silhavy *et al*., 2002; Voinnet *et al*., 1999) in three separate binary vectors under control of the enhanced cauliflower mosaic virus (CaMV) 35S promoter featuring a doubled enhancer. The antibody chains were targeted to the secretory pathway of the plant cells (Figure 2). For purification of the recombinant IgG, we used Protein G chromatography, which results in good enrichment (≥75% antibody purity) in one single step (Figure 2b and d). Recovery was typically around 70%, with the most significant loss (14%) during pH adjustment and centrifugation, whereas losses due to incomplete antibody adsorption to and elution off the affinity column accounted for ~8% and 5%, respectively. The product was stable for several months at 4 °C and subsequent gel‐filtration chromatography did not reveal any significant tendency of the purified antibody for aggregation or oligomerization at physiological pH (Figure 2d). Also, there was no noticeable change in antigen‐binding performance after storage (data not shown). Both *N. benthamiana* and *N. tabacum* were able to produce correctly processed and fully assembled chimeric antibody molecules with negligible signs of proteolytic degradation in the purified preparations.

The best reproducible yields in *N. benthamiana* leaves harvested 7 days postinfiltration (dpi), reached ~460 mg/kg. Yields after purification reached 329 mg of purified antibody per kg FW of leaf material with an average of 299 ± 35 mg/kg FW after purification (Figure 2c). Infiltrated *N. tabacum* plants showed much lower yields, reaching 119 mg/kg and averaging at 68 ± 41 mg/kg FW after purification (Figure 2c). These data represent the results from five independent experiments on each plant species. For follow‐up infiltration batches of *N. benthamiana* leaves, vacuum infiltration was used rather than manual syringe infiltration, and the antibody yields were within the same range.

### Plant‐derived MC10E7 retains the microcystin‐binding properties of its hybridoma‐derived counterpart

**Figure 3:**
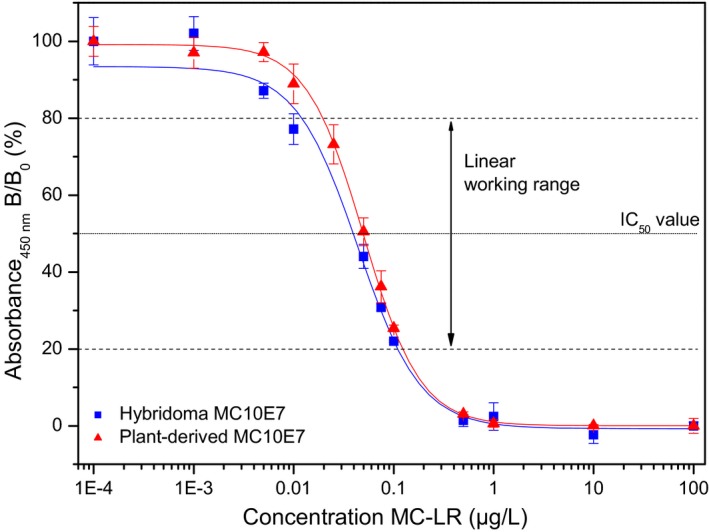
ELISA results obtained with purified antibodies on the same microtitre plate (*m* = 12 for plant‐produced antibody, *m* = 11 for monoclonal mouse‐antibody, *n* = 3). Error bars represent ±1 standard deviation about the mean.

The microcystin‐binding properties of the recombinant MC10E7 from plants were tested by indirect competitive ELISA, using an optimized set‐up in which the concentrations of the coating antigen and the antibody were kept as low as possible to achieve the highest sensitivity. Figure 3 shows the typical sigmoidal calibration curves of the original and plant‐derived versions of MC10E7, which were established with identical amounts of antibody. The assay was carried out for intra‐assay comparison on one microtitre plate, and all samples were measured in triplicate. The analytical working range (20%–80% inhibition) was 16.2–119.6 ng/L for hybridoma MC10E7 and 20.2–123.8 ng/L for plant‐derived MC10E7. The limit of detection (LOD) at a signal‐to‐noise (S/N) ratio of 3 was 10.9 ng/L for hybridoma MC10E7 and 12.5 ng/L for plant MC10E7. Finally, the inflection points (IC_50_ value), as indicators for the assay sensitivity, were 44.0 ± 4 ng/L for hybridoma MC10E7 and 50.0 ± 3 ng/L for plant MC10E7. In conclusion, the results were in very good agreement for both types of antibodies.

To evaluate the specificity of plant‐derived MC10E7, the cross‐reactivity (CR) of other [Arg4]‐microcystins (MC‐LW, MC‐RR and MC‐LF) was determined at the IC_50_ value of the calibration curve. The hybridoma‐produced MC10E7 showed a CR of 58% for MC‐RR and negligible values of <0.1% for MC‐LW and MC‐LF. Similarly, plant‐derived MC10E7 showed a CR of 62% for MC‐RR and <0.1% for MC‐LW and MC‐LF. This suggests that the L‐arginine residue in position (4) of microcystins is necessary for antibody binding.

In addition, comparative binding studies were carried out by surface plasmon resonance (SPR) spectroscopy. The association and dissociation rate constants of antibody–antigen (MC‐LR) interactions, which can be used to calculate antibody affinity, were determined using single cycle kinetics. This format eliminates the need for regeneration after every antibody injection (Frostell *et al*., 2013). For both antibodies, five concentrations (0.130, 0.261, 0.521, 1.040 and 2.030 nmol) were injected consecutively.

**Figure 4:**
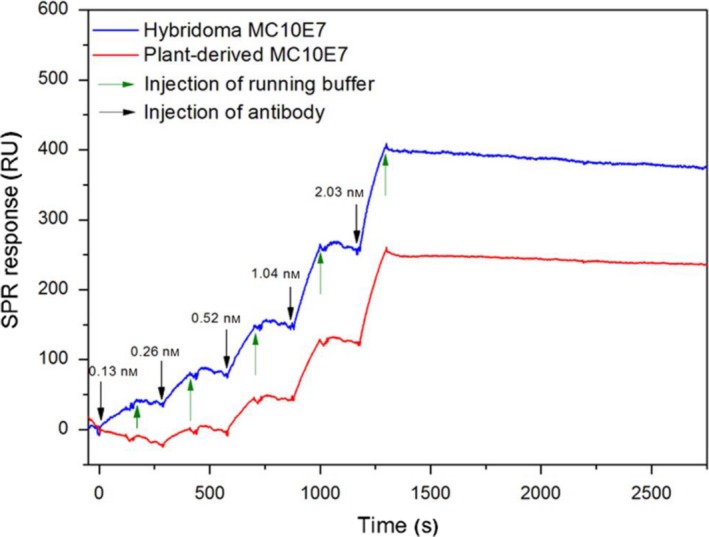
Single kinetic curves for hybridoma MC10E7 and plant‐derived MC10E7 using MC‐LR immobilized sensor chip CM5 and Biacore X100. Carrier solution: running buffer, flow speed 30 μL/min, five concentrations of individual mAbs between 0.13 nm and 2.03 nm; contact time: 2 min at 37 °C.

The performance of SPR at 25 °C yielded nondetectable dissociation of the antibodies from immobilized MC‐LR, stressing their extraordinary binding affinities. Interestingly, ELISA tests at a higher temperature of 37 °C had led to significantly higher IC_50_ values for both antibodies, that is 234 ± 17 ng/L for hybridoma MC10E7 and 143 ± 15 ng/L for plant‐derived MC10E7. The SPR experiment was therefore also carried out at 37 °C and yielded association rate constants (*k*_a_) of 3.27 × 10^6^ 1/Ms and 3.21 × 10^6^ 1/Ms and dissociation rate constants (*k*_d_) of 4.58 × 10^−5^ 1/s and 3.09 × 10^−3^ 1/s for hybridoma and plant‐derived MC10E7, respectively. The resulting dissociation constants (*K*_D_) were 1.40 × 10^−11^ m for hybridoma MC10E7 and 9.61 × 10^−10^ m for plant‐derived MC10E7; that is, the *K*_D_ values are proof of the extraordinary high binding affinity of both antibodies. Figure 4 shows representative single kinetic curves for both antibodies.

### A lateral flow dipstick immunoassay based on the plant‐made antibody is suitable for the assessment of water samples

**Figure 5:**
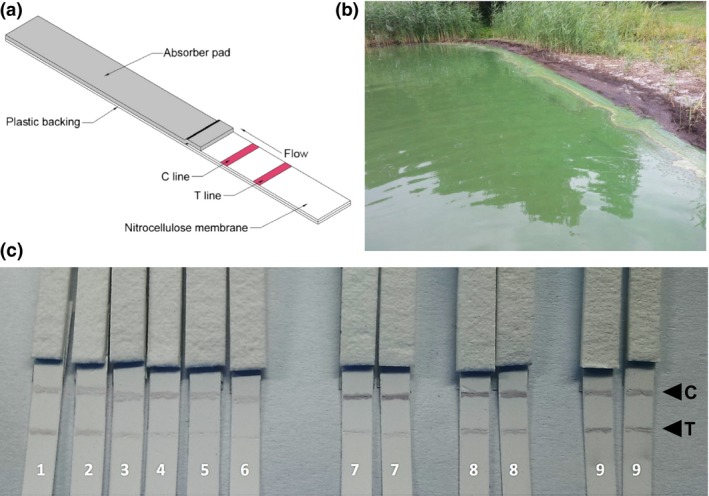
(a) Schematic illustration of the immunological dipstick. C line: control line with immobilized goat‐anti‐human IgG antibodies; T line: test line with immobilized MC‐LR‐BSA conjugate; (b) cyanobacterial bloom at lake *Rudower See*. (c) Typical photograph image of the detection of MC‐LR in standards and freshwater samples. Dipsticks no. 1–6: MC‐LR standards (blank, 0.1, 0.3, 0.5, 0.7, 0.9 μg/L); no. 7 and 8: *Rudower See* water, *Lenzen 1 and 2;* no. 9: river *Isar*.

The immunochromatography strip was based on a nitrocellulose membrane with plastic backing. The test(T) line and control(C) line were placed at 1.0 and 1.5 cm from lower end of the strip, respectively (Figure 5a). To ensure irreversible binding to the nitrocellulose membrane as a capture line, the small hapten (MC‐LR) was conjugated with bovine serum albumin (BSA) before loading to achieve surface binding and optimal antigen presentation. Samples were mixed with the MC10E7‐immobilized gold colloids and a semi‐quantitative visual evaluation of colour in the test and control lines was performed.

The concentrations of MC‐LR in the standard solutions were determined using the dipstick test. The colour intensity of the test line gradually decreased with increasing MC‐LR concentration. A significant decrease in the colour intensity was observed even at concentrations as low as 0.1 μg/L MC‐LR, and concentrations higher than 0.7 μg/L resulted in the almost complete disappearance of the T line. Considering the manual preparation and assembly of the test, the result suggests that the dipstick based on the plant‐derived MC10E7 is remarkably sensitive. Based on the data obtained from ELISA and SPR, comparable sensitivity can be expected with both plant‐derived and murine antibody.

In an initial proof‐of‐principle experiment, we tested two freshwater samples collected at different sites from a lake with evident cyanobacterial bloom (Rudower See, Figure 5b) and one sample from a river without recognizable algae contamination (Isar). Whereas the latter was negative, the lake water caused a significant loss of colour intensity in the T line (Figure 5c). Visual evaluation of the strips by naked eye allowed the detection of MC‐LR contamination at concentrations of 100–300 ng/L in the water.

## Discussion

### Antibody V‐gene rescue

The sequence of the variable region from a hybridoma‐derived antibody is usually determined by PCR, and rapid amplification of cDNA 5′‐ends (5′‐RACE) is routinely used to determine unknown mRNA sequences upstream of a gene‐specific primer, usually designed to anneal inside the heavy and light chain constant regions of a given antibody isotype. A primer‐binding site can be generated by homopolymer tailing (Frohman *et al*., 1988; Loh *et al*., 1989) or by adding an oligonucleotide of known sequence to the 5′‐end of either de‐capped mRNA prior to first‐strand cDNA synthesis (Clepet *et al*., 2004; Maruyama and Sugano, 1994) or to the cDNA after or during its synthesis (Chenchik *et al*., 1996; Edwards *et al*., 1991). We used the latter approach by exploiting intrinsic 3′‐terminal cytosine transferase and template‐switching abilities of a reverse transcriptase (Matz *et al*., 1999; Pinto and Lindblad, 2010).

Although the use of primers specific for the antibody constant region avoids some of the challenges attributed to primer design (Doenecke *et al*., 1997; Ruberti *et al*., 1994), this approach cannot completely exclude the amplification of incorrect antibody transcripts (Cabilly and Riggs, 1985; Carroll *et al*., 1988; Jiang *et al*., 1994; Krebber *et al*., 1997; Neumaier *et al*., 1990; Ostermeier and Michel, 1996; Vidarsson *et al*., 2001). This can be counteracted by obtaining supporting information from the peptide mass fingerprinting of the functional, hybridoma‐derived antibody using a technique such as matrix‐assisted laser desorption ionization time‐of‐flight mass spectrometry (MALDI‐TOF‐MS). This approach simplifies the design of gene‐specific primers and helps to identify incorrect transcripts that may be amplified (Essono *et al*., 2010).

### Production of the antimicrocystin recombinant antibody variants in heterologous expression systems

For nontherapeutic applications, in which antibodies are primarily required for their ability to recognize and bind specific antigens, the use of mammalian cells as production systems is often economically unfeasible, especially when large quantities are required and the cost of the antibody‐based products or devices must remain low (Frenzel *et al*., 2013). Because this antimicrocystin antibody is intended to be used as a toxin detector and scavenger in the context of water analysis and purification, large product quantities are needed as inexpensively as possible. Plant‐based and microbial expression systems are therefore attractive in this respect.

The *P. pastoris* expression system which is suitable for the production of soluble antibody fragments achieved yields of ~4 mg of purified scFv per litre of medium. However, the antigen‐binding activity of the scFv versions was more than 10‐fold lower than the hybridoma‐derived full‐length IgG. This may indicate the sensitivity of the antigen‐recognizing CDR loops of the MC10E7 Fab to subtle changes in the overall scaffold or the loss of rigidity, potentially leading to changes in molecular contacts with the antigen (Zdanov *et al*., 1994). The loss of binding activity when converting from full‐sized antibodies to the scFv format is not uncommon (Bird and Walker, 1991; Lorberboum‐Galski and Lazarovici, 2003).

We therefore set out to produce a full‐size chimeric version of the antimicrocystin antibody using sequences for human IgG1. Plants have proven to be suitable for the production of full‐size antibody formats, so we evaluated *N. benthamiana* and *N. tabacum* SR1 for small‐scale transient expression, with the opportunity to generate stable transgenic plants later if the initial tests were successful. Both species produced a fully assembled antibody with only minimal signs of proteolytic degradation, which sometimes occurs in both species due to the presence of plant proteases (Hehle *et al*., 2015; Niemer *et al*., 2014, 2016; Villani *et al*., 2009). The highest yields of the purified antibody were achieved in *N. benthamiana* leaves (299 ± 35 mg/kg FW) compared to 68 ± 41 mg/kg FW in *N. tabacum cv* Petite Havana SR1. A similar difference in performance between two *Nicotiana* species has been reported with human IL6 (Nausch *et al*., 2012), and these results agree with the more prevalent and widespread use of *N. benthamiana* for transient expression (Goodin *et al*., 2008). However, high variability in transient recombinant protein accumulation has also been shown between different *N. tabacum* cultivars, with some *tabacum* cultivars performing even better than *N. benthamiana* (Conley *et al*., 2011).

Prior to purification, we measured antibody levels of ~460 mg/kg in *N. benthamiana* leaves. Comparable antibody yields in nonreplicating transient systems have been achieved by others, and can be as high as 325 mg/kg FW (Sainsbury and Lomonossoff, 2008), 400 mg/kg (Saxena *et al*., 2011), 460 mg/kg (Garabagi *et al*., 2012) or in some cases 1.5 g/kg with average of 757 mg/kg FW (Vézina *et al*., 2009). The latter yield was achieved using syringe infiltration and with the assistance of another transgene silencing suppressor protein from *Potato virus Y* (HcPro) (Brigneti *et al*., 1998), but at larger scales using vacuum infiltration the average yield of the same IgG was 328 mg/kg FW. Even higher yields have been achieved with replicating plant viral vectors in which the viral RNA polymerases (RdRPs) produce large numbers of transcripts in each cell (Bendandi *et al*., 2010; Giritch *et al*., 2006; Huang *et al*., 2010).

Several recently published articles have addressed the techno‐economic principles of biomanufacturing facilities based on transient expression in *N. benthamiana* (Buyel and Fischer, 2012; Nandi *et al*., 2016; Tuse *et al*., 2014; Walwyn *et al*., 2015). Although the projected cost of goods sold (COGS) varies due to the effects of expression level, purification strategy and production capacity, there is generally a significant cost reduction compared to mammalian systems, and generally, the models agree very well when adjusted for production level, expression level and downstream recovery. The most recent model for a transient *Nicotiana*‐based platform for the production of a therapeutic monoclonal antibody arrives at a COGS of US$121/g including depreciation. This calculation considers a base case design scenario (production = 300 kg/y, yield = 1 g/kg FW and recovery = 65%) and takes into account all material and production costs (Nandi *et al*., 2016). The COGS increases sharply above US$1000/g when assuming a moderate production volume of only ~25 kg/y (Nandi *et al*., 2016). However, for technical/analytical antibodies, there are more relaxed requirements compared to pharmaceutical‐grade proteins in terms of purity and the removal of contaminants such as endotoxins and viruses, allowing the downstream processing and quality control costs to be cut significantly. The cost of preclinical studies and clinical trials can also be disregarded.

A techno‐economic analysis of the production of the nontherapeutic enzyme horseradish peroxidase by transient expression in *N. benthamiana* revealed that a yield of 240 mg HRP/kg. FW was economically feasible, and this is significantly lower than the yields of MC10E7 we achieved. Assuming a rather small production capacity of 5 kg purified HRP/year, their analysis indicated a high COGS of $1279/g. However, they showed that by doubling the biomass productivity and expression level, improving the downstream recovery from 54% to 63%, and increasing the production capacity to 20 kg HRP/year, the COGS was reduced to $611/g (Walwyn *et al*., 2015).

Dipsticks are reliable and inexpensive to produce because they are simple and the raw material costs per unit are low. In dipsticks based on a monoclonal antibody, the latter tends to be the most expensive component and further price reduction is therefore not possible. Currently, the commercial hybridoma‐derived mouse MC10E7 typically contributes to nearly half of the final cost of the assay (Tippkötter *et al*., 2009).

Production of the analogous plant‐derived antibody in the transient *Nicotiana*‐platform therefore offers a potential overall cost reduction. Costs could be further reduced by switching from transient expression to stable transgenic plants, which eliminates the need for bacterial cultures and infiltration. Once homozygous seed lines are developed, very little upstream process management is required, compensating for the often lower levels of expression (Garabagi *et al*., 2012). A direct comparison of antibody yields achieved by transient expression in *N. benthamiana* leaves and stable expression in *N. tabacum* plants (using the same pTRA‐based vector in both cases) revealed that selected transgenic lines were superior in yield (Teh *et al*., 2014). Further optimization could be achieved by replacing the laboratory‐scale purification strategy based on Protein G chromatography with a more economical procedure based on ion‐exchange chromatography.

A rapid lateral flow immunoassay for the detection of microcystin was developed using the plant‐derived antibody. Current analytical procedures usually involve on‐site sampling and the time‐delayed analysis of the toxin by protein phosphatase inhibition assay (PPIA), enzyme inhibition assay ELISA, HPLC or MALDI‐TOF‐MS (Fastner *et al*., 2001; Lawton *et al*., 1994; Metcalf *et al*., 2001; Rapala *et al*., 2002). ELISA methods have received most attention due to their high sensitivity and broad dynamic range (3–300 ng/L), but they take several hours to complete and the sample must be shipped to a laboratory (Pyo and Hahn, 2009). For the rapid on‐site assessments of MC‐LR contamination, cost‐effective dipstick assays would be more convenient.

Our lateral flow immunoassay was highly sensitive, with a noticeable loss of colour intensity on the T line at MC‐LR concentrations as low as 0.1 μg/L. Considering the manual preparation and assembly of the test, this result shows that the antibody‐based dipstick assay is remarkably sensitive. Even greater sensitivity below 100 ng/L and easier handling could be achieved using an automatic dispenser to print the proteins on the test strip, plus further optimization of the test protocol, and the use of a handheld reader to record the optical density or fluorescence intensity of the T and C line. To the best of our knowledge, the other lateral flow assays described in the literature thus far have not achieved the sensitivity for MC‐LR demonstrated by our dipstick assay (Lawton *et al*., 1990; Liu *et al*., 2014; Tippkötter *et al*., 2009). However, there are commercially available immunochromatographic tests with comparable sensitivity (http://www.abraxiskits.com/products/algal-toxins/#dipsticks). The excellent performance together with the possible cost reduction achieved using plant‐derived antibodies makes the production and marketing of user‐friendly test strips feasible and also offers the possibility to develop adsorbents for water purification requiring antibody production at very large scales.

## Conclusion

MC10E7 binds with high affinity and specificity to MC‐LR, the most toxic and most abundant of the microcystins. The V‐gene sequences were therefore rescued from this antibody and used to produce a chimeric derivative suitable for expression in plants, which are more stable and economically attractive than hybridoma lines. Our work confirms that plants are suitable for the rapid and cost‐effective production of sufficient amounts of functional recombinant MC10E7 for downstream applications such as the development of antibody‐based sensitive test systems for microcystin detection or antibody‐based scavengers for water treatment. The plant‐derived antibody was accordingly used to construct a highly sensitive lateral flow immunoassay (dipstick) suitable for the on‐site analysis of water samples.

## Experimental procedures

### Isolation of the heavy and light chain cDNAs

The antibody heavy and light chain cDNAs were isolated by the reverse transcription of total RNA from the MC10E7 hybridoma cell line (Institute of Hydrochemistry, Technical University Munich) followed by 5′‐RACE. The end adaptors required for second‐strand synthesis and amplification were attached during reverse transcription by exploiting the 3′‐terminal cytosine transferase and template‐switching properties of reverse transcriptase (Chenchik, *et al*., 1998). The template‐switching primer was designed with as little homology to the mouse genome as possible and it contained a 3′ C3‐spacer to prevent elongation by reverse transcriptase thus avoiding off‐target cDNA synthesis (Pinto and Lindblad, 2010). Primer sequences are provided in Appendix S1. First‐strand synthesis was carried out essentially as described by (Pinto and Lindblad, 2010), and 2 μL of the product was used for the second‐strand synthesis and 5′‐RACE reactions. Following 5′‐RACE, the reaction mixtures were separated on a 1.2% agarose gel and regions including the expected size (700–800 bp) were excised, ligated into the pJET1.2 blunt‐end vector and cloned in *Escherichia coli* strain DH10B. Individual colonies were screened by colony PCR using the ZTP‐F adapter primer and reverse primers annealing upstream of the kappa or gamma‐chain sequence (MIgGκ‐R30 or MIgGγ1‐R266, respectively). Sequences of at least five independent clones for each chain were aligned, and the consensus sequences were analysed. Specifically, peptide ions generated by the *in silico* digestion of the corresponding open reading frames were compared against those observed by peptide mass fingerprinting of the original MC10E7 antibody produced in hybridoma cells.

### Peptide mass fingerprinting of the hybridoma‐derived monoclonal antibody

A 5‐μg aliquot of MC10E7, obtained by ammonium sulphate precipitation of immunoglobulins from hybridoma fluid followed by dialysis and final purification using a goat‐anti‐mouse IgG‐Sepharose column, was fractionated by discontinuous SDS‐PAGE and protein bands corresponding to the HC and LC were visualized by staining with Coomassie Brilliant Blue R‐250 dye. The bands were excised separately, followed by in‐gel S‐carbamidomethylation and trypsinolysis (Shevchenko *et al*., 2007). For in‐solution digestion with thermolysin, MC10E7 was reduced in 6 m guanidine·HCl, 0.5 m Tris‐HCl pH8.5, 2.5 mm EDTA, 65 mm DTT and S‐carbamidomethylated by adding 2‐iodoacetamide to 50 mm. The modified polypeptide chains were precipitated in acetone and suspended in thermolysin digestion buffer (50 mm Tris‐HCl pH 8.0, 0.5 mm CaCl_2_). Thermolysin (V4001; Promega, Mannheim, DE) was added at an enzyme:substrate mass ratio of 1 : 30. Peptide samples in 5% formic acid and α‐cyano‐4‐hydroxycinnamic acid matrix were applied to a stainless‐steel MALDI plate. Mass spectra were acquired using an Autoflex Speed MALDI‐TOF/TOF instrument (Bruker) in both positive and negative ion mode.

### Construction of expression vectors

Expression vectors for the production of the chimeric IgG1 (MC10E7) were constructed based on pTRAkt HC and pTRAkt_LC binary vectors containing the human HC and LC constant domains and a murine IgG signal sequence (GenBank ID DQ407610) (Kapelski *et al*., 2015). The VH and VL regions of the isolated MC10E7 cDNA were amplified using primers AgeI‐VH_F (5′‐ATA CCG GTG TAC ATT CAG AGG TTA TGG TCG TTG AAT C‐3′) and SalI‐VH_R (5′‐CTC GTC GAC GCT GAT GAC ACT GTT ACA GAT G‐3′), or AgeI‐VL_F (5′‐ATA CCG GTG TAC ATT CAG ATG TTG TGA TGA CCC AGA C‐3′) and BsiWI‐VL_R (5′‐ GCG CGT ACG TAT GAT TTC CAG GTT GGT ACC‐3′), respectively. The amplified variable regions were inserted in‐frame between the signal sequence and corresponding constant domains present in pTRAkt_HC/LC plasmids as described by Kapelski *et al*. (2015).

### Transient expression of MC10E7 in *N. tabacum* and *N. benthamiana*

The binary constructs for the full‐size antibody (MC10E7) expression were transferred into *Agrobacterium tumefaciens* strain GV3101::pMP90RK and delivered to plant leaf cells by agroinfiltration (Bechtold and Pelletier, 1998). Another *A. tumefaciens* culture carrying a construct encoding the TBSV p19 silencing suppressor (Silhavy *et al*., 2002; Voinnet *et al*., 2003) was used for cotransformation. The cultures carrying the HC, LC and p19 vectors were mixed together at a ratio of 2 : 2 : 1, and the OD_600_ of the final mixture was adjusted to 0.4. The suspension was infiltrated into the abaxial side of *N. benthamiana* or *N. tabacum* L. cv. Petit Havana SR1 leaves manually with a syringe (Schob *et al*., 1997). Alternatively, *N. benthamiana* plants were infiltrated by submerging them in the bacterial suspension in a vacuum chamber and applying mild vacuum twice (~130 mbar) for 30–40 s followed by venting for additional 20–30 s (Leuzinger *et al*., 2013). Infiltrated plants were allowed to grow for 7 days at 20–22 °C, before postinfiltration leaves with large veins removed were processed immediately or frozen at –20 °C.

### Expression of MC10E7 scFv in *Pichia pastoris*

Yeast transformation and protein expression using the pPICZα vector were carried out according to the Invitrogen user manual. The X‐33 strain of *P. pastoris* was transformed with linearized vectors by electroporation. The best Mut^+^ clones of 10 for every scFv variant were selected in the course of a small‐scale expression experiment in test tubes. Optimal scFv yield and integrity in shake flasks were achieved using buffered BMMY medium (pH 7.2) at 15–17 °C with shaking at 180 r.p.m. The initial methanol concentration was 1% and was replenished to 0.5% every 16 h. Cultures were collected at 48 h postinduction.

### Protein purification

Transformed leaf material was ground to a fine powder in liquid nitrogen and mixed with half a volume of extraction buffer (PBS pH 7.4 containing 5 mm EDTA and 0.2 mm PMSF) before homogenizing the mixture by ultrasonication. Homogenates were incubated on a shaker for 1 h at 4 °C, and cell debris was removed by centrifugation at 9000 ***g*** for 20 min at 4 °C. The pH of the supernatant was then adjusted to pH 8.0, followed by another centrifugation step and filtration through a 1‐μm polypropylene filter. Further purification was carried out as previously described (Rademacher *et al*., 2008) with the exception that 1 mL of Protein G Sepharose 4 FF beads (GE Healthcare, Freiburg, Germany) was used for every 100 mL of the clarified extract. The protein content in fractions was determined using the Bradford assay and measuring UV_280_ absorbance with a calculated extinction coefficient (μ_a_) of 1.37 L/g/cm. Recovery was calculated as the percentage representation of the total amount of antibody eluted from the column compared to the soluble antibody content of the initial crude extract determined by ELISA. Gel‐filtration chromatography was carried out using Superdex 200 Increase 10/300 GL columns (GE Healthcare) with an isocratic flow rate of 0.75 mL/min (20 mm sodium phosphate pH 7.3, 140 mm NaCl). Proteins were detected using a BioLogic QuadTec UV–vis detector (Bio‐Rad, Hercules, CA, USA) at 280 nm. Molecular weight was calibrated using protein standards from the gel‐filtration LMW and HMW calibration kits (GE Healthcare) and with IgG1. The identity of the main peak was additionally verified by SDS‐PAGE of the corresponding fraction and by antigen‐binding assays.

To purify the scFv variants, clarified yeast medium was treated with ammonium sulphate to 45% saturation and left overnight at 4 °C to precipitate most of the scFv. Protein was redissolved and passed over a Profinity^™^ Ni^2+^‐charged IMAC resin (Bio‐Rad) following the manufacturer's instructions.

### SDS‐PAGE and immunoblots

Protein samples were denatured in Laemmli buffer containing 100 mm DTT and separated by discontinuous SDS‐PAGE. Proteins were either visualized using Coomassie Brilliant Blue R‐250 dye or transferred to a nitrocellulose membrane by semi‐dry transfer. Anti‐human IgG (H + L) AP conjugate (Promega) was used for detection. Antibody fragments were detected using a mouse anti‐c‐Myc polyclonal antibody followed by anti‐mouse IgG‐AP conjugate (Promega) in a similar fashion.

### Synthesis of the MC‐LR‐BSA conjugate

The MC‐LR‐BSA conjugate was synthesized as recently described (Neumann *et al*., 2016). In brief, sulfhydryl groups were introduced to BSA with *N*‐succinimidyl 3‐(acetylthio)‐propionate (SATP) and used to couple the MC‐LR via its dehydroalanine residue (the Michael acceptor) to the protein. After dialysis, the conjugate was characterized by MALDI‐TOF and revealed a coupling density of 3 MC‐LR molecules per molecule of protein.

### Microcystin‐LR enzyme‐linked immunosorbent assay (MC‐LR ELISA)

Buffers and solutions for the indirect competitive ELISA were prepared as previously described (Cervino *et al*., 2008) The ELISA tests were carried out in high binding microtitre plates (MTPs) which were coated with a MC‐LR‐BSA conjugate (50 ng/mL in coating buffer, 200 μL/well) overnight at 4 °C followed by automated washing step with washing buffer. For calibration curves, the prepared MC‐LR stock solution (1 mg/mL in 99% ACN, 1% DMSO) was diluted to final standard concentrations in the range 0.0001–100 μg/L in methanol/water (1/10, v/v). The competition was carried out by adding standard solution (100 μL/well) and anti‐MC‐LR antibody (5 ng/mL in PBS, 100 μL/well). HRP‐labelled antibodies (200 ng/mL anti‐human‐IgG‐HRP) (Sigma) or anti‐mouse‐IgG‐HRP (Vector Laboratories, Burlingame, CA) both in PBS, 200 μL/well) and colour development with substrate solution (200 μL/well) and stop solution (100 μL/well) were used for detection. Intra‐assay comparisons were made on one microtitre plate, and all samples were measured at least in triplicate. For evaluation of the results, a sigmoidal calibration curve was set up using Rodbard's four‐parameter function and the absorbance was plotted against the log concentration of MC‐LR standards.

### Cross‐reactivity

To investigate the selectivity of MC10E7 for other microcystins, standard dilutions of MC‐LW, MC‐LF and MC‐RR (all microcystins were purchased from Enzo Life Sciences, Lörrach, Germany) were prepared in methanol/water (1/10, v/v) to final concentrations in the range 0.001–1000 μg/L. Cross‐reactivities were calculated using the quotient of the IC_50_ values (concentration giving 50% inhibition) relative to MC‐LR in per cent.

### ELISA for plant‐produced antibody quantification

ELISA plates were coated with goat‐anti‐human γ‐chain IgG. Antibody‐containing samples were applied as serial dilutions. Serial dilutions of purified antibody with known concentrations over the range 0.1–100 ng/mL were used for calibration. Leaf extracts were also applied at several different dilutions. The secondary antibody was an anti‐human IgG‐AP conjugate. The plate was developed using p‐nitrophenyl phosphate substrate (Sigma) according to the manufacturer's recommendations.

### Surface plasmon resonance (SPR) spectroscopy

Experiments were conducted using a Biacore X100 instrument (GE Healthcare) with Biacore control software v1.1 and evaluation software v1.1. SPR measurements were taken using the indirect format on a CM5 chip. The CM5 sensor chip was activated outside the device with 50 μL of a 1 : 1 mixture EDC (0.4 m) and NHS (0.1 m), followed by washing the surface with HEPES (10 mm). For the antigen immobilization, 50 μL of a MC‐LR solution (0.1 mg/mL in 600 nm CTAB/10 mm HEPES) was added. For sensor chip deactivation, 50 μL of 1 m ethanolamine (pH 8.5/HCl) was added and incubated for 20 min. The kinetic constants were determined at 37 °C and a flow rate of 30μL/min using single cycles with injections of five antibody dilutions in running buffer (HBS EP+, pH 7.4) and each injection over 120 s. The dissociation time was set to 1500 s followed by a chip regeneration step. To determine the rate constants, a 1:1 binding model was applied using the Biacore X100 evaluation software.

### Development of a colloidal gold‐based lateral flow immunoassay

The colloidal gold probe was prepared according to Frens with slight modification as described previously (Frens, 1973; Neumann *et al*., 2016). An average particle diameter of 40 nm was obtained by mixing 50 mL water, 188 μL HAuCl_4_ (0.1 m in water) and 1.13 mL of aqueous sodium citrate solution (1% in water). For the coupling of plant‐derived MC10E7, 1 mL of the obtained colloidal gold solution was adjusted to pH 10 and incubated with plant‐derived MC10E7. BSA solution was used to block free binding sites on the colloidal gold. After centrifugation (3200 ***g***, 10 min, RT) and washing (0.05 mm PBS, pH 9.0, 0.1% PEG‐8000), the functionalized gold colloids were dispersed in washing solution and stored at 4 °C.

The immunochromatography strip comprised a nitrocellulose membrane with plastic backing (Immunopore RP; cat. no. 78356403; GE Healthcare) cut to 20 × 5 mm strips and an absorbent pad (Grade CF 5; cat. no. 29008181; GE Healthcare) cut to 30 × 5 mm strips. Using a 10‐μL glass syringe (Hamilton, Bonaduz, Switzerland), the test line (T line) was coated with the MC‐LR‐BSA conjugate (1 mg/mL in PBS) and the control line (C line) with goat‐anti‐human IgG, Fc specific, (2.1 mg/mL in PBS; cat. no. I2136; Sigma‐Aldrich), in each case using 1 μL of solution per cm on the membrane. The T line and C line were placed 1.0 and 1.5 cm from lower end of the strip, respectively. Then, the immobilized membrane was dried at room temperature for 1 h, followed by blocking nonspecific sites with BSA (1% in PBS). After three washes with PBS, the membrane was dried for 2 h at room temperature. The dipstick was assembled by pasting the absorption pad downstream from the C line by overlapping ~2 mm with the membrane, using an adhesive (Photo Tape, Hama GmbH, Monheim, Germany).

For the determination of the indicator range by naked eye detection, standard solutions at concentrations of 0, 0.1, 0.3, 0.5, 0.7, 0.9, 1.1, 1.3 and 1.5 μg/L were prepared by diluting MC‐LR stock solution (10 μg/mL in methanol; Sigma‐Aldrich) with distilled water. The indicator range was defined as the lowest concentration of MC‐LR which revealed an invisible test line. The test was performed on a low binding 96‐well microtitre plate as follows. A volume of 120 μL of sample was placed in the well, followed by 20 μL Tween‐20 (1% in water) and 35 μL of the antibody‐immobilized gold colloids (~3.3 μg). The suspension was pre‐incubated with shaking for 5 min. Then, the test strip was soaked into the suspension for ~10 min followed by the semi‐quantitative visual evaluation of the colour of the test and control lines.

For initial testing, field samples (0.5 L each) were collected on 30 August 2016 on lake *Rudower See* near Lenzen (north‐west of Province Brandenburg, Germany) and on 6 September 2016 on river *Isar* in Munich (Bavaria, Germany) at a depth of ~20–30 cm. Aliquots of each water sample (~10 mL) were filtered using SPARTAN syringe filters, hydrophilic, 30 cm (cat. no. 5824.2, Carl Roth, Karlsruhe, Germany) and then directly submitted to the wells of a microtitre plate for the dipstick test.

## Supporting information


**Figure S1** cDNA‐sequence of the original MC10E7 mAb from the mouse hybridoma cells.
**Figure S2** (a) SDS‐PAGE of the purified MC10E7 scFv (1.5 and 3 μg) from *Pichia pastoris* under reducing conditions. Staining with Coomassie Brilliant Blue R‐250; (b) western‐blot of the culture supernatant prior to purification. Probed with anti‐c‐*myc* pAb. (c) Cassette for yeast transformation (scFv).
**Appendix S1** Primers used for the Isolation of the heavy and light chain cDNAs.
