# Supplementary material for: Cloning and plant‐based production of antibody MC10E7 for a lateral flow immunoassay to detect [4‐arginine]microcystin in freshwater
Source: Plant Biotechnol J. 2017 Jun 5;16(1):27–38. doi: 10.1111/pbi.12746 (PMC5785354; doi:10.1111/pbi.12746)
Supplement: Supplementary file 1 — Figure S1 cDNA‐sequence of the original MC10E7 mAb from the mouse hybridoma cells. Figure S2 (a) SDS‐PAGE of the purified MC10E7 scFv (1.5 and 3 μg) from Pichia pastoris under reducing conditions. Staining with Coomassie Brilliant Blue R‐250; (b) western‐blot of the culture supernatant prior to purification. Probed with anti‐c‐myc pAb. (c) Cassette for yeast transformation (scFv). Appendix S1 Primers used for the Isolation of the heavy and light chain cDNAs. [file PBI-16-27-s001.pptx]

## Slide 1
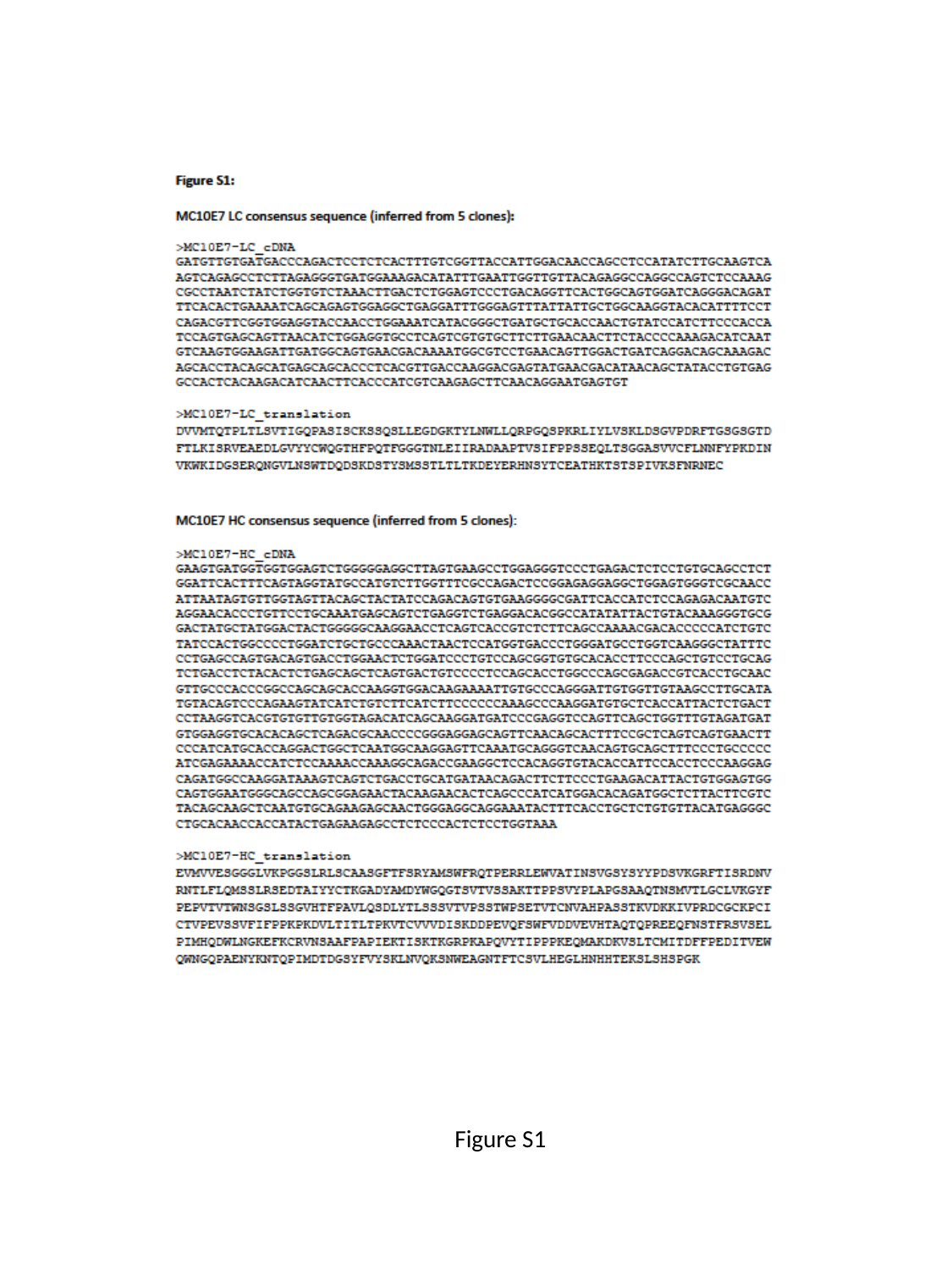

Figure S1
Figure S1

## Slide 2
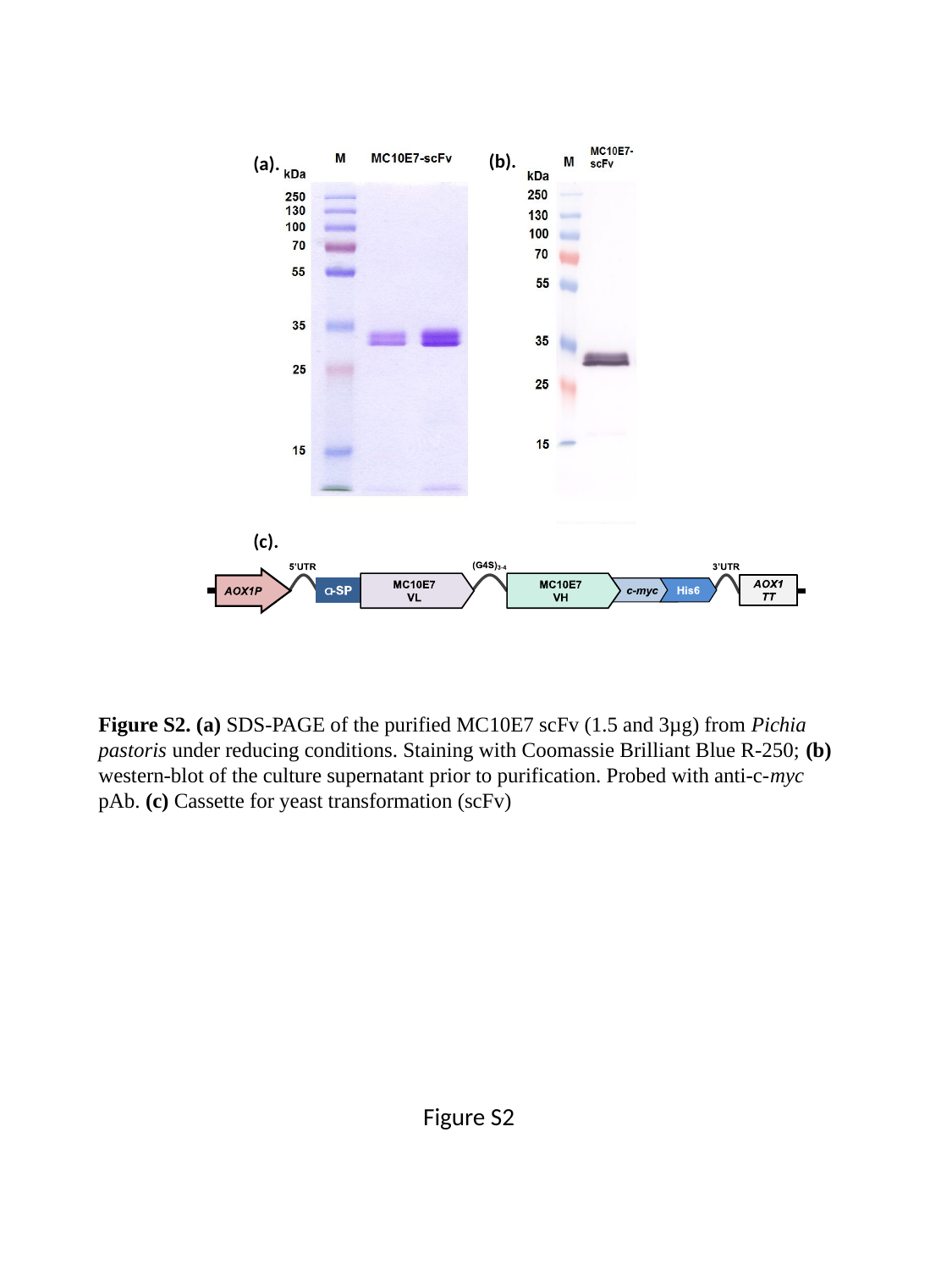

(b).
(a).
(c).
Figure S2. (a) SDS-PAGE of the purified MC10E7 scFv (1.5 and 3µg) from Pichia pastoris under reducing conditions. Staining with Coomassie Brilliant Blue R-250; (b) western-blot of the culture supernatant prior to purification. Probed with anti-c-myc pAb. (c) Cassette for yeast transformation (scFv)
Figure S2

## Slide 3
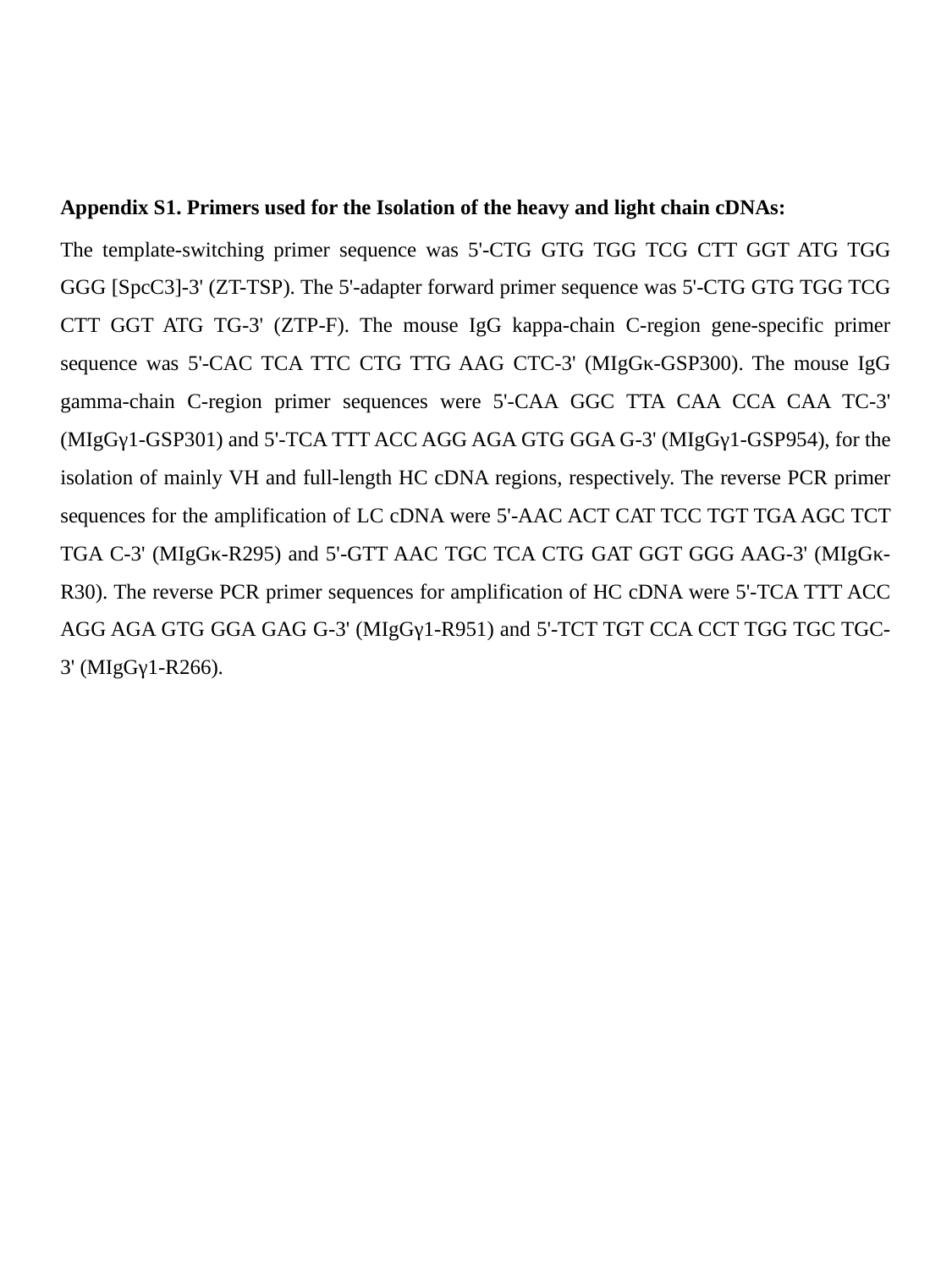

Appendix S1. Primers used for the Isolation of the heavy and light chain cDNAs:
The template-switching primer sequence was 5'-CTG GTG TGG TCG CTT GGT ATG TGG GGG [SpcC3]-3' (ZT-TSP). The 5'-adapter forward primer sequence was 5'-CTG GTG TGG TCG CTT GGT ATG TG-3' (ZTP-F). The mouse IgG kappa-chain C-region gene-specific primer sequence was 5'-CAC TCA TTC CTG TTG AAG CTC-3' (MIgGκ-GSP300). The mouse IgG gamma-chain C-region primer sequences were 5'-CAA GGC TTA CAA CCA CAA TC-3' (MIgGγ1-GSP301) and 5'-TCA TTT ACC AGG AGA GTG GGA G-3' (MIgGγ1-GSP954), for the isolation of mainly VH and full-length HC cDNA regions, respectively. The reverse PCR primer sequences for the amplification of LC cDNA were 5'-AAC ACT CAT TCC TGT TGA AGC TCT TGA C-3' (MIgGκ-R295) and 5'-GTT AAC TGC TCA CTG GAT GGT GGG AAG-3' (MIgGκ-R30). The reverse PCR primer sequences for amplification of HC cDNA were 5'-TCA TTT ACC AGG AGA GTG GGA GAG G-3' (MIgGγ1-R951) and 5'-TCT TGT CCA CCT TGG TGC TGC-3' (MIgGγ1-R266).
